# Small artery elasticity is decreased in patients with systemic lupus erythematosus without increased intima media thickness

**DOI:** 10.1186/ar3145

**Published:** 2010-09-28

**Authors:** Hans LA Nienhuis, Karina de Leeuw, Johan Bijzet, Jasper J van Doormaal, Arie M van Roon, Andries J Smit, Reindert Graaff, Cees GM Kallenberg, Marc Bijl

**Affiliations:** 1 Department of Rheumatology and Clinical Immunology, University Medical Center Groningen, University of Groningen, Hanzeplein 1, Groningen, 9713 GZ, The Netherlands; 2 Department of Internal Medicine, Division of Vascular diseases, University Medical Center Groningen, University of Groningen, Hanzeplein 1, Groningen, 9713 GZ, The Netherlands; 3 Department of Biomedical Engineering, University Medical Center Groningen, University of Groningen, Hanzeplein 1, Groningen, 9713 GZ, The Netherlands

## Abstract

**Introduction:**

The objectives of this study were to determine small arterial elasticity (SAE) in systemic lupus erythematosus (SLE) and to investigate its relationship with intima media thickness (IMT), accumulation of advanced glycation end products (AGEs), endothelial activation and inflammation.

**Methods:**

Thirty SLE patients with inactive disease and 30 age- and sex-matched healthy controls were included. Twenty patients with essential hypertension (EH) served as positive control. SAE was assessed by pulse-wave analysis using tonometric recordings of the radial artery. IMT of the carotid arteries was measured by ultrasound. AGE accumulation was assessed with an AGE-reader. Endothelial activation markers and C-reactive protein (CRP) were determined by enzyme-linked immunosorbent assay (ELISA).

**Results:**

SAE was decreased in SLE (*P *= 0.01) and further decreased in EH (*P *< 0.01) compared to healthy controls. IMT was increased in EH (*P *< 0.05), but not in SLE. AGE accumulation was increased in SLE (*P *< 0.05) and further increased in EH (*P *< 0.01) compared to healthy controls. Endothelial activation markers and CRP were increased in SLE but not in EH. SAE related to AGE accumulation (r = -0.370, *P *< 0.05), CRP (r = -0.429, *P *< 0.05) and creatinine clearance (r = 0.440, *P *< 0.05), but not to IMT and endothelial activation markers. In multivariate analysis SLE was an independent predictor of SAE.

**Conclusions:**

SAE is decreased in SLE patients without increased IMT, independently of traditional cardiovascular risk factors. Longitudinal studies are needed to investigate whether SAE, endothelial activation and AGE accumulation are early markers for cardiovascular disease in SLE.

## Introduction

Systemic lupus erythematosus (SLE) is associated with an increased prevalence of cardiovascular disease (CVD), due to accelerated atherosclerosis [1,2]. Traditional cardiovascular risk factors cannot fully explain the presence of accelerated atherosclerosis in these patients, suggesting that other factors are involved [3-5]. A potential non-traditional risk factor in these patients is formation and accumulation of advanced glycation end products (AGEs). We previously showed that accumulation of AGEs is increased in SLE patients and that AGE accumulation is related to intima media thickness (IMT), a surrogate marker for atherosclerosis [6].

Endothelial cell (EC) activation and dysfunction are among the first steps in atherogenesis [7]. Detection of these early and reversible events may be of clinical relevance, because it offers the possibility to intervene early in the process leading to atherosclerosis. The presence of EC activation can be assessed by measuring circulating levels of soluble vascular cell adhesion molecule-1 (VCAM-1), thrombomodulin (TM), and von Willebrand factor (vWf). EC dysfunction can be detected by several techniques of which flow mediated vasodilatation (FMD) is most commonly used. Another technique is pulse wave analysis (PWA) which measures large and small artery elasticity (LAE and SAE, respectively). SAE is decreased in high vascular risk conditions such as hypertension, diabetes mellitus and chronic kidney disease [8-10]. SAE is inversely related to IMT and in a retrospective study SAE was shown to be an independent predictor of cardiovascular events [11,12].

Based on these observations we hypothesized that SAE is decreased in SLE patients and that decreased SAE is related to non-traditional risk factors, including AGEs. For this reason, we determined artery elasticity in patients with SLE and related artery elasticity to EC activation, intima media thickness and traditional and non-traditional risk factors, including accumulation of AGEs.

## Materials and methods

### Subjects

Thirty consecutive patients (26 females, 4 males) fulfilling the American College of Rheumatology criteria for SLE [13], who attended the out-patient clinic of the University Medical Center Groningen, were included (Table 1). Aiming at markers for early detection of atherosclerosis, patients with a history of CVD, including ischemic heart disease (ICD-9 classification 410 to 414), cerebrovascular accidents or peripheral vascular disease, were excluded. Other exclusion criteria were pregnancy, diabetes mellitus, renal insufficiency (creatinine > 140 μmol/l) and active disease, defined as SLE Disease Activity Index (SLEDAI) > 4 [14]. Thirty age- and sex-matched healthy subjects were recruited as negative controls and 20 patients with still untreated essential hypertension (EH) were included as positive controls (Table 2). Hypertension was defined as a systolic blood pressure = 140 mmHg and/or a diastolic blood pressure = 90 mmHg, based on at least three measurements, and/or the use of antihypertensive drugs. Possible secondary causes of hypertension had to be excluded. The same exclusion criteria as for SLE patients applied to EH patients. All patients and controls were Caucasians, except for two SLE patients of Asian origin. The local research ethics committee gave approval for the study and written informed consent was obtained from each participant.

**Table 1:** Disease characteristics of SLE patients

	SLE (*n *= 30)
**ACR criteria, n (%)**	
**Malar rash**	13 (43%)
**Discoid rash**	6 (20%)
**Photosensitivity**	12 (40%)
**Oral ulcers**	9 (30%)
**Arthritis**	20 (66%)
**Serositis**	16 (53%)
**Renal disorder**	13 (43%)
**Neurological disorder**	1 (3%)
**Hematological disorder**	23 (77%)
**Immunological disorder**	26 (87%)
**Antinuclear antibody**	30 (100%)

**Duration of disease, months**	146 (49 to 209)
**SLEDAI**	4 (2 to 4)
**Anti-dsDNA, Farr**	20 (6 to 140)
**Complement C3, g/l**	0.89 (0.74 to 1.06)
**Complement C4, g/l**	0.12 (0.09 to 0.17)
**C-reactive protein, mg/l**	1.8 (0.5 to 4.5)
**Creatinine Clearance, ml/min**	106 (93 to 131)

**Prednisolone use, n (%)**	19 (63%)
**Dose, mg/day**	7.5 (5 to 10)
**Hydroxychloroquine use, n (%)**	15 (50%)
**Dose, mg/dag**	400 (400 to 400)
**Azathioprine use, n (%)**	11 (37%)
**Dose, mg/day**	100 (50 to 150)

Unless stated otherwise, data are expressed as median (25 to 75%). ACR, American College of Rheumatology; SLEDAI, SLE disease activity index; anti-dsDNA, anti-double stranded DNA.

**Table 2:** Traditional cardiovascular risk factors and vascular parameters of patients and healthy controls

	CTL (*n *= 30)	SLE (*n *= 30)	EH (*n *= 20)
**Age, years**	42 ± 11	41 ± 10	49 ± 15
**Female, n (%)**	26 (87%)	26 (87%)	12 (60%)
**BMI, kg/m^2^**	24 ± 2.6	25 ± 5.0	25 ± 3.7
**Blood pressure, mmHg**			
**Systolic**	120 ± 9	125 ± 17	151 ± 15**^##^
**Diastolic**	70 ± 6	72 ± 10	88 ± 9**^##^
**Dyslipidaemia, n (%)**	14 (47%)	15 (50%)	13 (65%)
**Smokers, n (%)**	3 (10%)	9 (30%)*	3 (15%)#
**Family history CVD, n**	5 (17%)	5 (17%)	7 (35%)
**HbA1c (%)**	5.4 ± 0.3	5.7 ± 0.7	5.3 ± 0.4
**Statins, n (%)**	0	1 (3%)	3 (15%)*
**Antihypertensives, n (%)**	0	9 (30%)**	0
**Beta-blockers**	0	4 (13%)*	0
**ACE inhibitors**	0	5 (17%)*	0
**Calcium antagonists**	0	1 (3%)	0
**Hypertension, n (%)**	0	9 (30%)**	20 (100%)**^##^

**SAE, ml/mmHg × 100**	8.1 ± 2.6	6.4 ± 2.7**	5.2 ± 3.8**
**AF, AU**	1.9 ± 0.4	2.1 ± 0.5*	2.3 ± 0.5**
**IMT, mm**			
**Mean Maximum**	0.72 (0.69 to 0.87)	0.71 (0.68 to 0.77)	0.89 (0.76 to 0.99)* ^##^
**Mean**	0.64 (0.59 to 0.70)	0.61 (0.57 to 0.66)	0.75 (0.67 to 0.86)** ^##^
**C-reactive protein, mg/l**	0.8 (0.4 to 1.5)	1.8 (0.5 to 4.5)*	1.7 (0.5 to 2.8)
**VCAM-1, ng/ml**	371 ± 101	548 ± 165**	393 ± 111^##^
**vWf, %**	67 (46 to 88)	130 (89 to 223)**	78 (50 to 177)^#^
**TM, ng/ml**	1.6 (1.2 to 1.7)	1.7 (1.2 to 2.3)	1.8 (1.5 to 2.6)*

Unless stated otherwise, data are expressed as mean ± standard deviation when normally distributed and as median (interquartile range) when non-normally distributed. ACE, angiotensin converting enzyme; AF, autofluorescence; AU, arbitrary units; BMI, body mass index; CrCl, creatinine clearance; CRP, C-reactive protein; CTL, healthy controls; CVD, cardiovascular disease; EH, essential hypertension patients; HbA1c, hemoglobulin A1c; IMT, intima media thickness; SAE, small artery elasticity; SLE, SLE patients; TM, thrombomodulin; VCAM-1, vascular cell adhesion molecule-1; vWf, von Willebrand factor. Patients versus controls: * *P *≤ 0.05, ** *P *≤ 0.01. EH patients versus SLE patients: ^# ^*P *≤ 0.05, ^## ^*P *≤ 0.01.

All traditional cardiovascular risk factors were assessed. Body mass index, smoking status, and family history of CVD (considered positive if first-degree relatives suffered from CVD before 60 years of age) were recorded. Dyslipideamia was defined as plasma cholesterol above 5.0 mmol/L and/or plasma low density lipoprotein (LDL) cholesterol above 3.0 mmol/L and/or triglycerides above 1.7 mmol/l and/or high density lipoprotein (HDL) cholesterol below 1.2 mmol/L in women and below 1.0 mmol/L in men and/or use of lipid lowering drugs [15]. Creatinine clearance was estimated using the Cockcroft-Gault formula. Use of prednisolone, azathioprine, and hydroxychloroquine was categorized as current and none, and daily dose was recorded. All measurements and blood sampling were performed after an overnight fast to exclude postprandial effects.

### Pulse-wave analysis

Arterial elasticity was assessed by pulse-wave analysis using the CR-2000 (Hypertension Diagnostics, Eagan, MN, USA). The CR-2000 records and analyses the blood pressure waveform data from the Arterial Pulse Pressure Sensor. SAE was estimated from a computerized pulse contour analysis using a Windkessel model [16]. Measurements were performed after 10 minutes of acclimatization in a temperature controlled room (22°C) with the subject in supine position. The arterial pulse pressure sensor was placed over the right and subsequently over the left radial artery. A blood pressure cuff was placed on the opposite arm to record blood pressure (BP). The average of three readings of BP and SAE of both the left and right arm were used for analysis. To assess reproducibility of this technique, 10 subjects of each group were studied by a single investigator on two separate occasions, with three weeks interspace. The intra-individual coefficient of variation was 11.2% for SAE.

### Assessment of AGE accumulation

Tissue AGE accumulation was assessed as skin autofluorescence (AF) on the ventral site of the lower arm with the AGE-Reader (DiagnOptics BV, Groningen, The Netherlands) [17]. In short, the AGE-Reader consists of a tabletop box containing a black light excitation light source (peak wavelength approximately 365 nm). Light emitted from the skin is measured with an integrated spectrometer. Measurement is fully automated and takes approximately 30 seconds to perform, giving an average value over 50 individual scans. The measurement was performed at non-lesional skin. Skin AF is calculated by dividing the mean value of the emitted light intensity per nm between 420 and 600 nm by the mean value of the excitation light intensity per nm between 300 and 420 nm, expressed as arbitrary units (AU). Repeated measurements on one day in controls and diabetic patients showed an overall Altman error percentage of five percent [17].

### Measurement of intima media thickness

Details of the method, as used in this study, have been described by de Groot *et al*. [18]. IMT was determined at the common carotid artery (CCA), the bulb and the internal carotid artery (ICA) at left and right sites using an Acuson 128XP ultrasound system with 7 MHz linear array transducers (Acuson Corp., Silicon Valley, California, USA). A B-mode image was obtained after which a probe was positioned perpendicular to the far wall, showing an intima-media complex over approximately one centimeter. Mean IMT (the mean of the segment studied) and the maximum IMT (the highest IMT value found among the segment studied) were determined. As endpoint we used the mean of the mean (mean IMT) and the mean of the maximum (mean to max IMT) of the far wall IMT of the six imaged carotid segments. From studies on repeatability, the error of variation in measurement is calculated as 0.03 mm for the carotid far wall IMT.

### Blood analyses

Fasting cholesterol, HDL, LDL, triglycerides, creatinine, glycated haemoglobin (HbA1c) and C-reactive protein (CRP) levels were measured by routine techniques. In patients, levels of complement C3 and C4 were measured by nephelometry, and levels of antibodies to double stranded DNA (dsDNA) by 125I Farr assay. Additionally, serum and plasma samples were stored at -20°C for determination of levels of markers of EC activation. Serum levels of vascular cell adhesion molecule-1 (VCAM-1) (R&D Systems, Abingdon, UK) and thrombomodulin (TM) (Diaclone, Besancon, France) were measured according to the manufacturer's instructions. Levels of von Willebrand factor (vWf) were determined using an in-house ELISA as described [19].

### Statistical methods

Power analysis revealed that 30 subjects in each group had to be included to detect a difference in SAE of 2.0 ml/mmHg × 100 with a standard deviation of 2.8 at a significance level of 0.05 with a power of 80%.

Except when stated otherwise, values are expressed as mean ± standard deviation when normally distributed and as median (interquartile range) when non-normally distributed. Comparisons between groups were made by Mann-Whitney tests or two-sample *t*-test for continuous variables and by chi-square analysis for categorical variables. Univariate correlations were assessed by Pearson's correlation coefficient, when variables were normally distributed. Otherwise, Spearman's correlation coefficient was used.

To assess influence of traditional cardiovascular risk factors on SAE, univariate linear regression and multivariate linear regression with backward inclusion of variables was performed. Variables that had a *P*-value < 0.05 in univariate analysis together with variables that significantly differed between healthy controls and SLE patients were included in the model. The probability of F for removal was 0.10.

All analyses were performed using SPSS 16.0 (SPSS Inc., Chicago, IL, USA). A two-sided *P*-value < 0.05 was considered to indicate statistical significance.

## Results

### Characteristics of patients and controls

Characteristics of patients and age- and sex-matched controls are presented in Tables 1 and 2. Concerning traditional cardiovascular risk factors, more SLE patients were smoking compared to healthy controls (30% vs 10%, respectively) and SLE patients more often used antihypertensive drugs compared to healthy controls (30% vs 0%, respectively). Patients with essential hypertension had increased diastolic and systolic blood pressure and more often used statins compared to healthy controls.

### Small Artery Elasticity

SAE was decreased in SLE patients (*P *= 0.01) and EH patients (*P *< 0.01) compared to healthy controls (Table 2). Since the increased prevalence of hypertension and smoking among SLE patients could have influenced results, we reanalysed data after exclusion of subject with hypertension and smokers. After exclusion of both subjects with hypertension and smokers, SAE remained significantly decreased in SLE patients (*n *= 14) compared to controls (*n *= 27, *P *= 0.021).

### Intima media thickness

Mean to max IMT and mean IMT did not differ between SLE patients and healthy controls, whereas both the mean to max IMT and mean IMT were increased in EH patients compared to healthy controls (*P *< 0.05 and *P *= 0.01, respectively) (Table 2).

### Accumulation of advanced glycation end products

AGE accumulation was increased in SLE patients (*P *= 0.02) and in EH patients (*P *< 0.001) compared to healthy controls (Table 2).

### Markers of EC activation and inflammation

Markers of EC activation are presented in Table 2. Levels of vWf and sVCAM-1 were elevated in SLE patients compared to healthy controls (*P *< 0.001 and *P *< 0.001, respectively) but not in EH patients. Levels of TM did not differ between SLE patients and healthy controls, whereas levels of TM were increased in EH patients compared to healthy controls (*P *= 0.04). Levels of CRP were increased in SLE patients (*P *< 0.05) but not in EH patients.

### Influence of traditional cardiovascular riskfactors on SAE

Univariate and multivariate analysis was performed to assess whether the difference in SAE between SLE patients and healthy controls could be explained by traditional cardiovascular risk factors. Factors that related to SAE in univariate analysis and factors that significantly differed between SLE patients and healthy controls (Table 2) were included in multivariate analysis. This analysis revealed that age, gender, smoking, and SLE are independently associated with SAE (Table 3). In addition, we assessed whether SAE and other vascular parameters differed between SLE patients with and SLE patients without hypertension, no significant differences were found (Table 4).

**Table 3:** Univariate and multivariate linear regression analysis for SAE in SLE patients and healthy controls (CTL)

	Univariate analysis		**Multivariate analysis (R**^**2 **^**= 0.524)**	
	
	B (95% CI)	*P*-value	B (95% CI)	*P*-value
Age (yr)	-0.119 (-0.182 to -0.056)	< 0.001	-0.126 (-0.178 to 0.074)	< 0.001
Gender^a^	-2.357 (-4.392 to -0.323)	0.024	-2.067 (-3.656 to -0.477)	0.012
BMI (kg/m2)	0.092 (-0.094 to -0.278)	0.324	**	
Blood pressure (mmHg)				
Systolic	-0.019 (-0.073 to 0.035)	0.486	**	
Diastolic	-0.065 (-0.154 to 0.024)	0.151	**	
Antihypertensives use^b^	0.201 (-1.636 to 2.037)	0.828	*	
Dyslipidaemia^c^	-0.645 (-2.106 to 0.816)	0.381	**	
Smoking^d^	-2.542 (-4.240 to -0.844)	0.004	-2.186 (-3.589 to -0.782)	0.003
Family history CVD^e^	0.667 (-1.285 to 2.619)	0.497	**	
HbA1c	-0.597 (-2.895 to 1.702)	0.596	**	
SLE or CTL^f^	-0.916 (-1552 to -0.279)	0.005	-1.425 (-2.549 to - 0.301)	0.014

B refers to influence on SAE.

* Variable was not selected during multivariate regression analysis.

**Variable was not tested in multivariate regression analysis because of a *P*-value > 0.05 in univariate regression analysis and/or no significant difference between between healthy controls and SLE patients.

^a^: male were defined as 0, female as 1, ^b^: non-hypertensive subjects were defined as 0, hypertentive subjects as 1, ^c^: no dyslipidaemia was defined as 0, dyslipidaemia as 1, ^d^: non-smokers were defined as 0, smokers as 1, ^e^: negative family history was defined as 0, positive family as 1, ^f^: healthy controls were defined as 0, SLE patients were defined as 1.

**Table 4:** Differences in vascular parameters between SLE patients with and without hypertension

	CTL (*n *= 0)	SLE - hypertension (*n *= 21)	SLE + hypertension (*n *= 9)
**SAE, ml/mmHg × 100**	8.1 ± 2.6	6.4 ± 2.1*	6.4 ± 4.0
**AF, AU**	1.9 ± 0.4	2.1 ± 0.5*	2.2 ± 0.6
**IMT, mm**			
**Mean Maximum**	0.72 (0.69 to 0.87)	0.70 (0.68 to 0.76)	0.73 (0.60 to 0.98)
**Mean**	0.64 (0.59 to 0.70)	0.61 (0.58 to 0.65)	0.62 (0.55 to 0.81)
**VCAM-1, ng/ml**	371 ± 101	534 ± 159**	578 ± 182**
**vWf, %**	67 (46 to 88)	123 (89 to 189)**	156 (83 to 397)**
**TM, ng/ml**	1.6 (1.2 to 1.7)	1.8 (1.4 to 2.6)	1.3 (0.6 to 1.8)

Unless stated otherwise, data are expressed as mean ± standard deviation when normally distributed and as median (interquartile range) when non-normally distributed. AF, autofluorescence; AU, arbitrary units; CTL, healthy controls; MT, intima media thickness; SAE, small artery elasticity; SLE, SLE patients; TM, thrombomodulin; VCAM-1, vascular cell adhesion molecule-1; vWf, von Willebrand factor. Patients versus controls: * *P *≤ 0.05, ** *P *≤ 0.01. There were no significant differences between SLE patients without hypertension and SLE patients with hypertension.

### Relations between SAE, IMT, AGE accumulation and disease related factors

Univariate analysis performed on data of SLE patients and healthy controls together revealed inverse correlations between SAE and AGE accumulation (r = -0.336, *P *= 0.009), CRP (*r *= -0.307, *P *= 0.022) and creatinine clearance (r = 0.376, *P *= 0.004). In SLE patients, SAE inversely correlated to AGE accumulation (r = -0.370, *P *= 0.044). Concerning disease related factors, SAE was positively correlated to creatinine clearance (r = 0.440, P = 0.017) and inversely correlated to CRP (r = -0.429, P = 0.025). No significant correlations were found between SAE and IMT.

## Discussion

In searching for early markers for atherosclerosis we evaluated SAE in relation to EC activation, inflammation, intima media thickness and accumulation of AGEs in patients with SLE without overt CVD and related our findings to patients at risk for premature atherosclerosis, which are those with untreated essential hypertension (EH).

First, we found that SAE is decreased in SLE patients as was the case in our positive control group consisting of EH patients. More importantly and in contrast to EH patients, this decrease was found in SLE patients with EC activation but without increased IMT and could not be explained by the presence of traditional cardiovascular risk factors. Second, we confirmed our previous finding that AGE accumulation is increased in SLE patients [6] and found that AGE accumulation is inversely related to SAE.

In contrast to others [20], we assessed SAE in patients without overt cardiovascular disease. In accordance with the latter, IMT was not increased in our SLE patients, supporting the assumption that SAE is an early marker of atherosclerosis. SAE might reflect EC function. Several studies have reported a correlation between SAE and flow-mediated vasodilatation (FMD), the traditional test of EC function [10,21]. Our results are in agreement with other studies showing EC dysfunction in patients with SLE using FMD [22,23]. Although FMD is a useful research tool to assess endothelial function, its poor reproducibility and its demands on the examiner and subjects limit the use of FMD in clinical practice [24]. PWA is well-tolerated and requires less time and experience of the examiner. We showed that reproducibility of this technique is acceptable, especially when natural variation in blood pressure (CV: 4.8% for systolic BP and 5.7% for diastolic BP), which is related to vascular function, is taken into account. In addition, van Doornum *et al*. showed that SAE is a more sensitive measure of vascular dysfunction than FMD in rheumatoid arthritis (RA) [21].

However, whether SAE is solely a measure of EC function is questionable. Besides EC function, functional and structural changes are likely to be determinants of artery elasticity as well. Duprez *et al*.showed that SAE is inversely related to IMT, suggesting that IMT is a determinant of SAE or visa-versa [11]. Although the physiological meaning of Windkessel-derived elasticity values remains unclear, they can still be used as biomarkers of arterial dysfunction [25].

IMT was significantly increased in EH patients, but did not differ between SLE patients and healthy controls. As our SLE patients did have decreased SAE without having an increased IMT, we suggest that SAE is already decreased prior to intima media thickening. Previous studies showed an increased IMT in SLE patients [3,4,26]. Discordance between this study and previous studies might be due to differences in age, disease duration and selection for patients without CVD. However, IMT values of our patients closely resemble those found by Roman *et al *in one of the largest studies to date on atherosclerosis in SLE [5]. Their SLE patients even had a decreased IMT compared to matched controls while patients had significantly more carotid plaques. This indicates that IMT, although considered a reliable marker for diffuse atherosclerosis, might not be a sensitive marker for localised atherosclerotic lesions. To decrease the possibility of missing localised atherosclerotic lesions we measured IMT at six different segments and in addition to mean IMT we also calculated the maximum IMT; however, no differences were found between SLE patients and controls.

Multivariate analysis revealed that SLE is an independent predictor of decreased SAE. Several disease-related factors might be involved, including chronic inflammation. Levels of CRP were increased in our SLE patients, even though the disease was inactive. Moreover, CRP inversely correlated with SAE, suggesting that inflammation contributes to vascular dysfunction. This finding is in accordance with several other studies reporting relations between CRP and indices of vascular function [27-29]. It has been suggested that CRP itself contributes to the development of atherosclerosis, as it exerts direct proinflammatory effects on endothelial cells [30]. However, a recent study shows that polymorphisms in the CRP gene, leading to increased CRP levels, are not associated with an increased risk of vascular disease [31]. Therefore, the role of CRP as causal factor in the development of atherosclerosis remains controversial.

The positive correlation between creatinine clearance and SAE suggests that renal function is also involved in vascular dysfunction and atherogenesis. This is further supported by the inverse correlation we found between IMT and creatinine clearance in SLE patients (data not shown). There is growing evidence that relatively minor renal abnormalities such as a slightly reduced GFR or microalbuminuria, even within the normal range, may be associated with increased risk of CVD. One of the principal pathophysiological mechanisms that have been proposed to link renal insufficiency to atherosclerosis is EC dysfunction caused by dyslipidemia, increased blood pressure, oxidative stress and low grade inflammation [32].

Oxidative stress and low grade inflammation, together with decreased clearance of AGE precursors, might lead to increased formation and accumulation of AGEs. AGEs are a class of compounds resulting from non-enzymatic glycation of proteins, lipids or nucleic acids under influence of oxidative stress. AGEs accumulate continuously on long-lived proteins with aging, and are present in inflamed tissue, such as rheumatoid synovia and atherosclerotic blood vessels [33]. We confirmed our previous findings that AGE accumulation is increased in non-lesional skin of SLE patients [6] and found that AGE accumulation is inversely related to SAE. Three general mechanisms may explain this relation. First, the cross-linking of AGEs with proteins in the extracellular matrix results in a decrease of blood vessel elasticity. Second, intracellular AGE formation may alter cellular function. Third, AGEs may modulate the function of cells by interaction with and activation of the receptor for AGEs (RAGE) and other receptors [34].

## Conclusions

In conclusion, vascular dysfunction, as shown by decreased SAE, is present in quiescent SLE patients without atherosclerosis. This decrease is independent of traditional cardiovascular risk factors, but is related to the presence of SLE. Disease related factors, such as inflammation, impaired renal function and accumulation of AGEs might be contributing to this vascular dysfunction. Measurement of SAE seems a clinically applicable method to detect early vascular changes in SLE.

## Abbreviations

ACR: American College of Rheumatology; ACE: angiotensin converting enzyme; AF: autofluorescence; AGEs: advanced glycation end products; AU: arbitrary units; BMI: body mass index; BP: blood pressure; CCA: Common Carotid Artery; CrCl: creatinine clearance; CRP: C-reactive protein; CTL: healthy controls; CVD: cardiovascular disease; dsDNA: double stranded deoxyribonucleic acid; EC: endothelial cell; EH: essential hypertension; ELISA: enzyme-linked immunosorbent assay; FMD: flow mediated vasodilatation; HbA1c: glycated haemoglobin; HDL: high density lipoprotein; ICA: internal carotid artery; ICD: International Classification of Diseases; IMT: intima media thickness; LAE: large artery elasticity; LDL: low density lipoprotein; PWA: pulse wave analysis; SAE: small artery elasticity; SLE: systemic lupus erythematosus; SLEDAI: systemic lupus erythematosus disease activity index; TM: thrombomodulin; VCAM-1: vascular cell adhesion molecule-1; vWf: von Willebrand factor.

## Competing interests

R. Graaff and A.J. Smit are both founders of DiagnOptics B.V., The Netherlands, which manufactures AGE readers for assessing skin autofluorescence.

## Authors' contributions

HLAN contributed to concept and design, performance of vascular and laboratory measurements, acquisition, analysis and interpretation of data, and drafting the article. KdL contributed to concept and design, interpretation of data and revising the article. JB contributed to performance laboratory measurements and revising the article. JJvD contributed to inclusion EH patients, interpretation of data and revising the article. AMvR contributed to concept and design, coordination of vascular measurement and revising the article. AJS contributed to concept and design, inclusion EH patients, interpretation of data and revising the article. RG contributed to validation of AF measurements and revising the article. CGMK contributed to concept and design, interpretation of data and revising the article. MB contributed to concept and design, inclusion SLE patients, interpretation of data and drafting the article.
